# Matrix Metalloproteinases: How Much Can They Do?

**DOI:** 10.3390/ijms21082678

**Published:** 2020-04-12

**Authors:** Magnus S. Ågren, Ulrich auf dem Keller

**Affiliations:** 1 Digestive Disease Center and Copenhagen Wound Healing Center, Bispebjerg Hospital, University of Copenhagen, 2400 Copenhagen, Denmark; 2 Department of Clinical Medicine, Faculty of Health and Medical Sciences, University of Copenhagen, 2400 Copenhagen, Denmark; 3 Department of Biotechnology and Biomedicine, Technical University of Denmark, 2800 Kongens Lyngby, Denmark; uadk@dtu.dk

**Keywords:** extracellular matrix, inflammation, wound healing, cytokines, proteinases, interstitial collagens

## Abstract

Zinc-dependent matrix metalloproteinases (MMPs) belong to metzincins that comprise not only 23 human MMPs but also other metalloproteinases, such as 21 human ADAMs (a disintegrin and metalloproteinase domain) and 19 secreted ADAMTSs (a disintegrin and metalloproteinase thrombospondin domain). The many setbacks from the clinical trials of broad-spectrum MMP inhibitors for cancer indications in the late 1990s emphasized the extreme complexity of the participation of these proteolytic enzymes in biology. This editorial mini-review summarizes the Special Issue, which includes four review articles and 10 original articles that highlight the versatile roles of MMPs, ADAMs, and ADAMTSs, in normal physiology as well as in neoplastic and destructive processes in tissue. In addition, we briefly discuss the unambiguous involvement of MMPs in wound healing.

More than half a century ago, Gross and Lapière discovered a true collagenase, which was the first vertebrate matrix metalloproteinase (MMP) responsible for the resorption of the tail in the metamorphosing tadpole [1]. We now know that vertebrate collagenase belongs to the metzincins, which is a clan of metalloendopeptidases found in all living organisms [2,3]. The metzincins, with their third ligand being histidine or aspartate in the active site, comprise not only the MMP family, which has 23 members in humans [4], but also other metalloproteinases, such as adamlysins or reprolysins, including ADAMs (a disintegrin and metalloproteinase domain; 21 members in humans) and ADAMTSs (a disintegrin and metalloproteinase thrombospondin domain), consisting of 19 secreted enzymes and at least 7 ADAMTS-like proteins that are devoid of catalytic activity [5,6], astacins (e.g., meprins, bone morphogenetic protein-1), leishmanolysins, serralysins, and snapalysins [2].

Research activities that followed the discovery by Gross and Lapière focused in the beginning on the critical role of these proteinases in extracellular matrix (ECM) remodeling in homeostatic balance and imbalance [7]. Thus, it turned out that MMPs are necessary for multiple and diverse physiological processes, such as reproduction, morphogenesis, embryonic development, bone remodeling, angiogenesis, and tissue repair, but they can also contribute to tissue destruction during cancer development and spreading and in arthritis/osteoarthritis and fibrotic diseases. The association of pathologies with MMP overexpression was also the impetus for the intense exploration of synthetic MMP inhibitors (MMPIs), especially those targeting cancer diseases, in the mid and late 1990s [8,9]. The results of randomized controlled trials were overwhelmingly disappointing for small-molecule MMPIs, due to their poor oral bioavailability, lack of efficacy, dose-limiting toxicities, and undesired musculoskeletal side effects [10]. These first-generation synthetic MMPIs targeted broadly by mimicking MMPs’ natural substrate (usually collagen) [8], but later innumerable substrates were identified [11]. Unfortunately, these early MMPIs also inactivate proteinases unrelated to the disease but necessary for one or more physiological processes [8]; the importance of ADAMs and ADAMTSs was unknown at the time. Today, there is consensus that MMPs, ADAMs, and ADAMTSs function in many cell-signaling pathways, in which they are probably even more important than in ECM remodeling [11,12]. In parallel with our increased understanding of the diverse biological roles of these proteinases, the current goal is the development of highly selective inhibitors [8,13] although there is still no approved MMPI available. The only exception is low-dose oral doxycycline (Periostat^®^), which was approved in 1998 as an adjunct for the treatment of adult periodontitis. The mechanism of the beneficial doxycycline at subantimicrobial doses (20 mg b.i.d.) involves inhibiting collagenase (MMP-8) activity [14].

The content of this Special Issue highlights the multiple biological functions of these proteolytic enzymes and includes four review articles [4,15,16,17] and 10 original articles [18,19,20,21,22,23,24,25,26,27]. The articles address the role of MMPs, ADAMs, and ADAMTSs, in normal physiology as well as in neoplastic and tissue destructive processes.

Reflecting their pleiotropic activities, MMPs are closely associated with direct and indirect cell communication by modifying cell adhesion via integrin interactions and by activating or inactivating cytokines/chemokines or other signaling biomolecules and their cognate receptors. The specific roles of MMPs in these complicated signaling networks are highlighted by Young et al. [17].

A specific example of MMP-dependent cytokine signaling was elucidated by Sammel et al. [27]. They showed that not only ADAM-10 but also ADAM-17 and several other MMPs can shed the interleukin (IL)-11 receptor to induce transsignaling, a process where soluble receptor fragments interact with the ligand to act on cells not responsive to the ligand alone. Functional redundancy ensures a robust response, even in the absence of individual members of the shedding machinery. Although the current study remains at the level of cell culture and overexpression systems, it warrants further investigation of this elaborate proteinase network in animal models and in samples from patients with disturbances in individual components.

Another example of the importance of MMPs as proteinases with essential functions that have evolved within robust networks with redundant activities is presented by Rogerson et al. in their studies on ADAMTS [26]. In their research study in genetically manipulated mice, Rogerson et al. identified ADAMTS-9 as a novel aggrecanase that, in the absence of the aggrecanolytic ADAMTS-4 and ADAMTS-5, is highly increased in its abundance and might assume the critical functions of ADAMTS-4 and ADAMTS-5 in normal skeletal development [26]. ADAMTS-4 and ADAMTS-5 are thought to contribute to osteoarthritis by degrading the proteoglycan aggrecan in articular cartilage [28]. ADAMTSs are highly conserved in mice and humans, but it remains to be explored if similar redundancies also contribute to human pathologies.

The way in which membrane-tethered MMPs can interact with soluble members of the MMP family was demonstrated by Albrechtsen et al. at the University of Copenhagen [18]. By identifying basigin, an inducer of soluble MMPs, as a novel shed substrate of ADAM-12, they revealed a new function of the proteinase within the MMP network. This mechanistic insight has the potential to help devising novel strategies for inhibiting aberrant MMP activity in carcinogenesis, although in vivo validation is required.

Yip et al. [16] thoroughly reviewed the literature on MT4-MMP (MMP-17), another membrane-anchored MMP that has only been poorly studied [4]. MT4-MMP is one of the two members (the other one is MT6-MMP) of the family that is tethered to the membrane by a glycosylphosphatidylinositol anchor and shows specific activity in processing only a few ECM components. These appear to be important in many diseases, particularly in several types of cancer, which again demonstrates the strong need to better understand every member of the MMP family as a potential target for therapy.

Several studies in this Special Issue corroborate findings that show that MMPs have to be tightly controlled to prevent detrimental activity in disease. This might also be achieved by posttranslational modification (PTM) of the proteinase or the substrates. Many PTMs in MMPs and their target proteins have been identified, but their dynamic relationships are still poorly understood. In a review article, Madzharova et al. [4] summarize the current knowledge of the PTM (glycosylation, phosphorylation, and glycosaminoglycans)-mediated control of MMP activity and review the technologies used to study this topic.

As an additional layer of the control of MMP activity, soluble MMPs can also activate each other, e.g., by propeptide removal. For instance, the stromelysin MMP-3 is incapable of cleaving triple-helical fibrillar collagens but increases collagenolysis via the activation of collagenases [29]. Mirastschijski et al. [24] has convincingly demonstrated the profound effect of the lack of MMP-3 on tumor necrosis factor-α (TNF-α)-initiated collagenolysis in the skin of MMP-3-deficient mice, a finding that supports the indirect pathological role of MMP-3 in skin collagen catabolism through the activation of the human collagenase MMP-1 [30]. The authors did not demonstrate a direct link between murine MMP-13 and collagenase activity.

Oku et al. [25] have studied the effect of inflammation on cancer metastasis and the involvement of matrix metalloproteinase-9 (MMP-9). They used exogenous TNF-α to mimic the in vivo conditions and found that TNF-α upregulated MMP-9 at the transcriptional and translational levels in gastric cancer and mesothelial cell lines. The peritoneal barrier was modeled in vitro by mesothelial cells grown on a basement membrane matrix. TNF-α increased cancer cell invasion of the mesothelium via a process that was inhibited by MMP-9 gene silencing but not by MMP-2 gene silencing and was rescued by the addition of MMP-9 (Figure 1).

Huber et al. [22] set out to elucidate the cellular mechanisms involved in the differential effect of high-molecular-weight heparin (HMWH) compared with that of other anticoagulants on MMP-9 blood levels. For this purpose, monocytic and T and B lymphocytic cell lines were cocultured. The researchers demonstrated that HMWH increased IL-16 and sICAM-1 secretion by T lymphocytes, which in turn increased IL-8 and MMP-9 production by monocytes (Figure 2).

To investigate the emerging functions of MMPs as immune modulators, Bates et al. [19] mimicked the influence of inflammatory processes initiated by *Porphyromonas gingivalis* in vitro, causing periodontal destruction. They developed a 3-cell coculture model that includes monocyte-derived dendritic cells, CD4+ T lymphocytes, and primary gingival keratinocytes. MMP-7 and MMP-12 production differed significantly between the single cell cultures and the cocultures.

Dreschers et al. [20] addressed the clinical problem of premature delivery due to intrauterine infection. They hypothesized that detrimental persistence of inflammation is caused by reduced apoptosis of neonatal monocytes following phagocytosis of the infectious agent, e.g., *Escherichia coli*. The reduced apoptosis of infected neonatal monocytes compared with that of infected adult monocytes was attributed to increased shedding of CD95L by MMP-9. TACE (ADAM-17) was not involved in this process. One drawback of the study was the use of the general MMP inhibitor chlorhexidine; thus, the involvement of other MMPs could not be ruled out [31].

Tuberculosis remains a serious infectious disease, causing 2 million deaths every year at a global level. In a comprehensive review of MMP involvement in tuberculosis, Rohlwink et al. [15] summarize the current knowledge of the functions of MMPs in tuberculosis infections in both the lung and the brain. They outline the critical activities of MMPs in ECM degradation and pathogen release in the lung as well as in modulating immune responses. The use of various MMPIs in preclinical models of pulmonary and central nervous system tuberculosis is also reviewed. The ambiguous roles of MMPs in disease progression, particularly in childhood tuberculous meningitis, underscore the need for an increased understanding of how to balance MMP activity in treatment strategies.

Neuropathic pain is very difficult to treat, and there is an unmet medical need for effective therapeutic approaches. Kwan et al. [23] evaluated the effect of MMP-2/MMP-9 inhibition on neuropathic pain (Figure 3). They used an orally bioavailable MMP-2/MMP-9 inhibitor (AQU-118) in the spinal nerve ligation rodent model of neuropathic pain and discovered a novel relationship between elevated MMP-2 mRNA expression levels and caspase-3-mediated cell death, once again highlighting the complex interactions of MMPs with other proteinases within the proteinase network.

Snake venom metalloproteinases belong to the adamlysins, and their antithrombotic effects are well-known [32]. Huang et al. [21] tested the therapeutic effect of the metalloproteinase SP, which was isolated and purified from the venom of a moccasin snake (*Agkistrodon acutus*), in animal models of induced thrombosis and pulmonary embolism. The protein prolonged the coagulation time and inhibited platelet aggregation and thrombosis. Mechanistically, metalloproteinase SP cleaved the α, β, and γ chains of fibrinogen.

During wound healing, MMPs are involved in multiple cellular, molecular, and biochemical processes [33]. To decipher the role of MMPs, we used synthetic nondiscriminative MMPIs in animal and human acute injury wound-healing models [34,35,36,37,38,39]. The main conclusion of these studies is that neoepithelium formation is severely impaired by blocking MMP activity, while, paradoxically, increased collagen deposition in skin or peritoneal or intestinal wounds does not result from inhibiting MMP activities [34,35,36,37]. Without the protective epithelium, there is an increased susceptibility to infection. This presents a therapeutic challenge for nonhealing chronic cutaneous wounds, which often present with excessive MMP activity [33,40,41]: therapy should block the activity of pathogenic MMPs but not affect the activity of the MMPs required for reepithelialization [39]. The MMPs responsible for reepithelialization are unknown, but mouse models indicate that MMP-9 is one candidate [42], and in vitro studies suggest MMP-1 and MMP-7 as important candidates as well [43,44]. MMP-10 is highly upregulated in keratinocytes of the migrating tip in wounds with dermal involvement [39,45] and can cleave several cell adhesion and bioactive proteins [46]. Nonetheless, MMP-10-deficient mice did not show severe deficits in epithelial healing [47], indicating that the role of redundancy in the interconnected MMP network needs to be explored. Another clinical example is the regeneration of the mucosal epithelium of anastomotic wounds after resection of diseased colorectal tissue. While they are beneficial for normal anastomotic wound healing [9,48], broad-spectrum MMPI therapies are detrimental to anastomotic wound repair under complicated conditions [49]. Under these conditions, nonselective MMPIs severely delay epithelial coverage resulting in increased invasion by pathogenic microorganisms, abscess formation, and anastomosis insufficiencies [49]. Additionally, inhibition of the antimicrobial MMP-12 may contribute to weakening of the host defense [50]. The use of more selective MMPIs or local delivery of the MMPI to avoid systemic side effects may be a solution [37,51].

In conclusion, the present Special Issue has shed some light on the complex functions of MMPs, ADAMs, and ADAMTSs, in physiological and pathological processes. The new experimental and collected data that are provided here add to the current knowledge. The identification of novel substrates has been critical to the recent advances of the MMP research field [52], and novel high-throughput degradomics technologies have been instrumental in extending the classical view on MMPs as simple tissue degraders to precise signaling scissors modulating complex immune responses. The use of these promising technologies together with valid disease models and clinical studies has the potential to translate into effective therapeutic modulators inhibiting detrimental MMP activities and enhancing their beneficial effects as targets and antitargets in diseases [12,53].

## Figures and Tables

**Figure 1:**
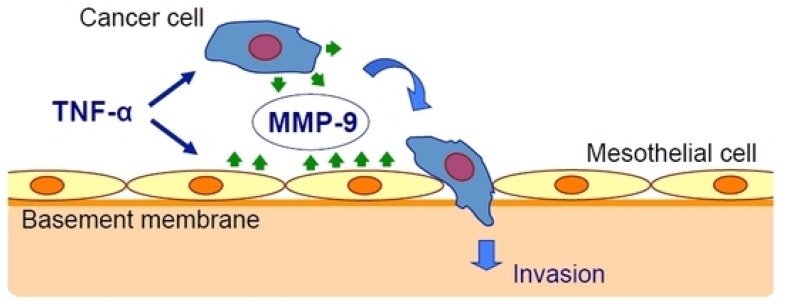
Tumor necrosis factor-α (TNF-α) stimulates gastric carcinoma cells and peritoneal mesothelial cells to secrete matrix metalloproteinase-9 (MMP-9), which promotes cancer cell invasion [25].

**Figure 2:**
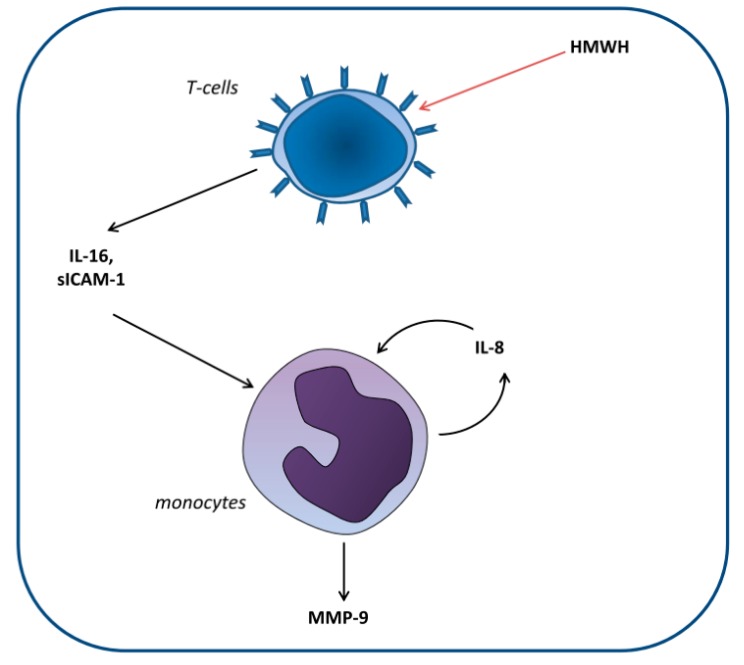
Proposed model of the induction of matrix metalloproteinase-9 (MMP-9) production in monocytes by high-molecular-weight heparin (HMWH)-treated T cells. T cells secrete the mediators IL-16 and sICAM-1, which induce monocytic IL-8 production. Together, these factors induce continuous IL-8 secretion as well as enhanced MMP-9 production by monocytes [22].

**Figure 3:**
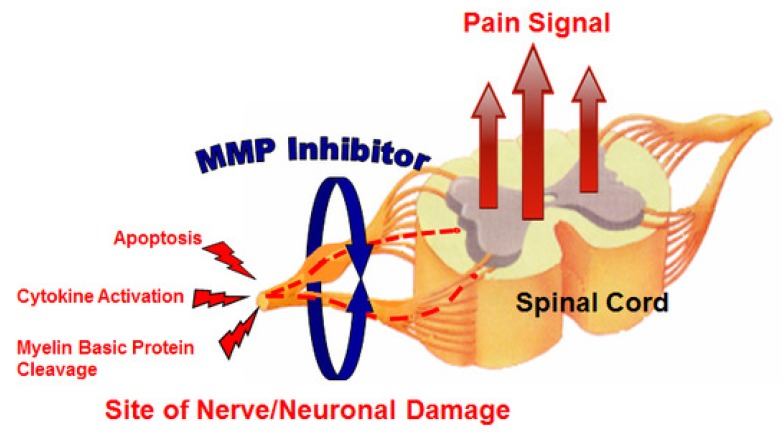
MMP inhibitor (MMPI) targets (increased apoptosis, increased cytokine activation, and decreased myelin basic protein levels) in neuropathic pain [23].

## Abbreviations

ADAM	A disintegrin and metalloproteinase domain
ADAMTS	A disintegrin and metalloproteinase thrombospondin domain
ECM	Extracellular matrix
HMWH	High-molecular-weight heparin
IL	Interleukin
MMP	Matrix metalloproteinase
MMPI	Matrix metalloproteinase inhibitor
PTM	Posttranslational modification
sICAM-1	Soluble intercellular adhesion molecule
TACE	Tumor necrosis factor-α converting enzyme
TNF-α	Tumor necrosis factor-α
